# Multiple serous cavity effusion screening based on smear images using vision transformer

**DOI:** 10.1038/s41598-024-58151-2

**Published:** 2024-03-28

**Authors:** Chunbao Wang, Xiangyu Wang, Zeyu Gao, Caihong Ran, Chen Li, Caixia Ding

**Affiliations:** 1 Department of Pathology, The First Affiliated Hospital of Xi’an Jiaotong University, Xi’an, 710061 China; 2 School of Computer Science and Technology, Xi’an Jiaotong University, Xi’an, 710049 China; 3 CRUK Cambridge Centre, University of Cambridge, Cambridge, CB2 0RE, UK; 4 Department of Pathology, Ngari Prefecture People’s Hospital, Ngari of Tibet, 859000 China; 5 Department of Pathology, Shaanxi Provincial Tumor Hospital, Xi’an, 710061 China

**Keywords:** Cancer screening, Machine learning

## Abstract

Serous cavity effusion is a prevalent pathological condition encountered in clinical settings. Fluid samples obtained from these effusions are vital for diagnostic and therapeutic purposes. Traditionally, cytological examination of smears is a common method for diagnosing serous cavity effusion, renowned for its convenience. However, this technique presents limitations that can compromise its efficiency and diagnostic accuracy. This study aims to overcome these challenges and introduce an improved method for the precise detection of malignant cells in serous cavity effusions. We have developed a transformer-based classification framework, specifically employing the vision transformer (ViT) model, to fulfill this objective. Our research involved collecting smear images and corresponding cytological reports from 161 patients who underwent serous cavity drainage. We meticulously annotated 4836 patches from these images, identifying regions with and without malignant cells, thus creating a unique dataset for smear image classification. The findings of our study reveal that deep learning models, particularly the ViT model, exhibit remarkable accuracy in classifying patches as malignant or non-malignant. The ViT model achieved an impressive area under the receiver operating characteristic curve (AUROC) of 0.99, surpassing the performance of the convolutional neural network (CNN) model, which recorded an AUROC of 0.86. Additionally, we validated our models using an external cohort of 127 patients. The ViT model sustained its high-level screening performance, achieving an AUROC of 0.98 at the patient level, compared to the CNN model’s AUROC of 0.84. The visualization of our ViT models confirmed their capability to precisely identify regions containing malignant cells in multiple serous cavity effusion smear images. In summary, our study demonstrates the potential of deep learning models, particularly the ViT model, in automating the screening process for serous cavity effusions. These models offer significant assistance to cytologists in enhancing diagnostic accuracy and efficiency. The ViT model stands out for its advanced self-attention mechanism, making it exceptionally suitable for tasks that necessitate detailed analysis of small, sparsely distributed targets like cellular clusters in serous cavity effusions.

## Introduction

Serous cavity effusion refers to the pathologic accumulation of body fluids, which is most commonly seen in pleural, abdominal, and pericardial cavities^1^. An accurate diagnosis is essential for identifying the underlying cause, determining the appropriate treatment, and predicting patient outcomes. One indispensable diagnostic tool for examining serous cavity effusions is the cytological examination, which is particularly useful for screening benign or malignant cases^2^. Cytological smears are easy to prepare, convenient, fast, and relatively inexpensive^1,3^. However, obtaining an accurate diagnosis can be challenging for cytologists. The large field of view of cytological smears can make it difficult to identify small targets with sparse distribution, leading to missed diagnoses^3^. Additionally, distinguishing between proliferative mesothelial cells and cancer cells can be challenging, especially for junior cytologists^4^. It is worth noting that the misdiagnosis rate of serous cavity effusions by cytologists is as high as 30%^5^. Such a high misdiagnosis rate not only poses risks to patient health but also leads to potential delays in initiating appropriate treatments^5^. Moreover, detailed cytology screening tasks are time-consuming and labor-intensive for cytologists. Studies have shown that it can take 30–40 min for doctors to screen a single smear^3^. This is particularly challenging in developing countries where there is a shortage of professional cytologists, making it difficult to complete diagnoses in a timely and accurate manner. Given these challenges, exploring the capabilities of artificial intelligence technologies in assisting with the diagnosis of serous cavity effusions is necessary. Such technologies, when properly developed and implemented, may offer a promising solution for improving the accuracy and efficiency of diagnosis.

In the early stages of artificial intelligence research, machine learning techniques like support vector machines (SVMs) and random forests were utilized in medical image analysis, predating the era of deep learning^6^. These early methodologies typically required careful feature extraction and engineering to be effective. However, they often encountered difficulties with complex, high-dimensional data or in identifying subtle but crucial diagnostic features in medical images^7^—a common challenge in the field of medical diagnostics.

Recently, deep learning architectures have emerged as a transformative force in diagnostic applications, from retinal disease identification^8^ to the detection of various cancers^9^. Notably, in cytological screening, methods such as graph convolutional networks (GCNs) have been employed to effectively interpret complex omics data, aiding in the identification of cervical^10–12^ and urothelial cancers^13,14^, as well as evaluating pleural effusion^15^. In a similar vein, pretrained lightweight deep-learning (PLDL) methods have been adapted for the clinical-level screening of Parkinson’s disease (PD) in older adults, utilizing two-fold training on hand-drawn wave/spiral patterns to distinguish between healthy and PD subjects with high accuracy^16^.

A seminal work on pleural effusion has demonstrated that convolutional neural networks (CNNs) can outperform junior cytologists in accuracy when identifying malignant lesions^15^. This breakthrough suggests that deep learning could serve as a robust adjunct tool for diagnostic support, especially in medical settings where access to expert cytologists is limited. Despite its promise, this study still has several limitations. Firstly, due to the inherent limitations of convolutional neural networks (CNNs), their receptive fields are restricted in scope, impeding their ability to effectively capture expansive features and the interrelations among them, which are often crucial for pleural effusion screening. Secondly, the dataset’s origin from a singular medical institution introduces a significant caveat at the data level, casting doubt on the model’s ability to maintain its performance across diverse clinical datasets. Lastly, this work only concentrates on pleural effusions and overlooks the fact that effusions can manifest in various serous cavities, each with its unique diagnostic challenges^1^. Furthermore, the morphological heterogeneity of malignant cells, attributable to their distinct histological origins^17^, further complicates the differentiation between proliferative mesothelial cells and neoplastic entities^4^. This heterogeneity underscores the necessity for developing powerful deep-learning frameworks that can be rigorously validated across a spectrum of serous cavity effusions, thereby broadening the horizons for clinical diagnostic accuracy and efficiency.

In the realm of deep learning for medical image analysis, CNN architectures are widely used for their proficiency in capturing spatial dependencies and identifying patterns across various imaging modalities. However, CNNs have inherent limitations in extracting fine details of small objects and in discerning complex relationships between different regions in images, which can reduce their effectiveness in certain diagnostic scenarios. In contrast, models based on transformer architectures, known for their success in natural language processing due to self-attention mechanisms, are gaining recognition. The introduction of the vision transformer (ViT)^18^ has been a significant development, signaling a shift towards purely transformer-based approaches for image classification. By processing sequences of image patches, this approach leverages the inherent strengths of attention mechanisms to effectively pinpoint crucial areas within images.The self-attention mechanism of the ViT model allows it to focus on specific parts of an input image that are more informative for the classification task. In the context of serous cavity effusion, where small cellular clusters are critical for accurate diagnosis, this mechanism enables the model to dynamically highlight and analyze these small targets within the broader context of the image. Unlike convolutional approaches that may dilute the importance of small, localized features through pooling layers, the ViT model can maintain high-resolution attention throughout the model. This results in a more precise analysis of critical features, which is particularly beneficial for identifying small clusters of malignant cells. Continuing research in areas like object detection and semantic segmentation has further demonstrated the transformer’s versatility. It efficiently captures a wide range of features, from global to local, thereby enhancing its applicability and effectiveness in more complex tasks^19–21^.

In the specific context of serous cavity effusion diagnosis via smear image analysis, the task of pinpointing sparse targets and discerning nuanced cellular features within an expansive visual field constitutes a formidable challenge. Traditional CNN frameworks may falter in their ability to detect the subtle nuances of these fluid collections, whereas a transformer-based model is posited to excel in discerning fine-grained patterns^22^. Thus, an exploration into the deployment of transformer-based models for serous cavity effusion diagnosis is posited to potentially enhance the precision and efficiency of clinical diagnostics. Our investigation delves into the efficacy of transformer-based models for the identification of malignant cells within conventional smears of various serous-cavity fluids, encompassing pleural, ascites, and pericardial effusions. A comparative analysis reveals that a ViT model markedly surpasses a conventional CNN model (ResNet-50)^23^ in screening performance for serous cavity effusions, attaining a caliber commensurate with clinical application. To substantiate the effectiveness of the proposed methodology, a dual-pronged experimental framework was employed, utilizing a novel Smear Image Classification (SIC) dataset alongside an External Patient Cohort (EPC-SIC). The SIC dataset, comprising annotations from 161 patients, was meticulously curated at the First Affiliated Hospital of Xi’an Jiaotong University. Concurrently, the EPC-SIC dataset was assembled from 127 cases at the Shaanxi Provincial Cancer Hospital.

The primary objective of this paper is to develop a highly accurate and efficient transformer-based classification framework for the automated screening of malignant cells in serous-cavity fluid smear images. The main contributions of this paper are as follows: (1) the curation of a novel, multi-center annotated dataset, with extensive experimentation conducted across multiple serous cavities. (2) Adaptation and optimization of the ViT model to address the specific challenges posed by cytological smear images. (3) Demonstrating through empirical evidence that transformer-based models can outperform traditional CNNs in this field, with important implications for clinical practice. In conclusion, this study highlights the transformative impact of transformer-based models in medical image analysis, particularly in enhancing the accuracy and efficiency of detecting malignant cells in serous cavity effusions. Our results support the adoption of deep learning approaches, especially transformer-based models, as a valuable tool to aid cytologists in diagnosis, ultimately improving patient care outcomes.

The rest of the paper is organized as follows. “Results”, presents the outcomes of our experiments, including a comparative analysis of the ViT and CNN models at both patch-level and patient-level classifications. It also discusses the model’s performance on an external validation cohort. “Discussion”, interprets the results in the context of current medical practices, explores the implications of our findings, and the potential of transformer-based models in clinical settings, and addresses the limitations and future directions of our research. “Methods”, describes the data collection and annotation process, the development of the transformer-based classification framework, and the specific methodologies employed for the analysis of cytological smear images.

## Results

### Patch level classification performance on SIC dataset

In the conclusion of our investigation, we systematically developed and compared several classification paradigms, including the vision transformer (ViT), ResNet-50, Vgg-16, and Fundus-DeepNet, for the purpose of screening serous cavity effusion cases within patient cohorts. Our methodology entailed a rigorous evaluation through five-fold cross-validation on the annotated segments of the Smear Image Classification (SIC) dataset. The results, encapsulated in Table 1, substantiate the superior accuracy and robustness of the transformer-based approach over the conventional CNN models.

Notably, the ViT model achieved an impressive accuracy of 96.8%, significantly outstripping the performance of the CNN models, with ResNet-50 at 87.3%, Fundus-DeepNet at 88.7%, and Vgg-16 trailing at 83.9%. The lower standard deviation of 2.2% for the ViT model, in contrast to ResNet-50’s 3.2%, Fundus-DeepNet’s 2.4%, and Vgg-16’s 1.7%, further corroborates the transformer’s consistent performance across varied data subsets. This consistency highlights the model’s robustness, an essential characteristic for clinical applications where stability across different patient cases is paramount.

**Figure 1:**
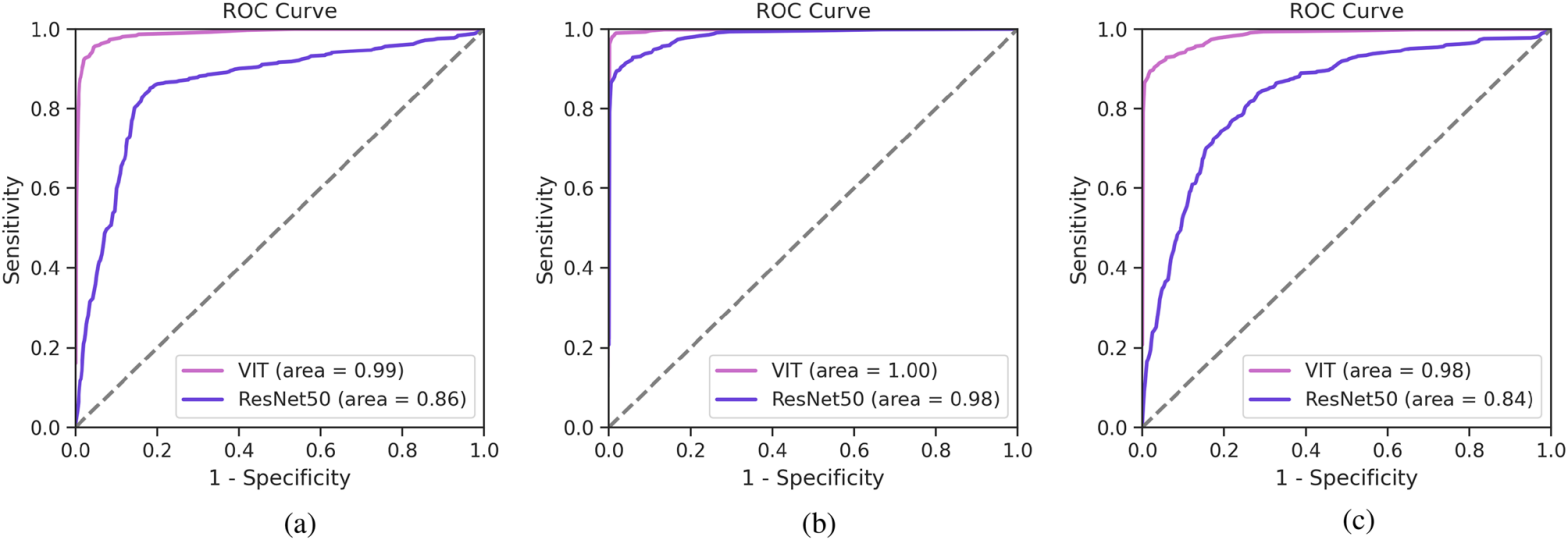
AUROC of each structure in the SIC and EPC-SIC dataset.

The ROC curves for patch-level classification, shown in Fig. 1a, reveal the vision transformer (ViT) model’s high discriminative ability when identifying malignant cells in serous cavity effusion smears. With an AUROC score of 0.99 on the internal dataset, the ViT model demonstrates an exceptional level of accuracy at this granular level. This is significant for the cytological examination of smears, which traditionally faces challenges in efficiency and diagnostic precision.

**Table 1:** Patch-level classification performance (%) of the ViT and CNN models on the SIC dataset.

Model	Fold 1	Fold 2	Fold 3	Fold 4	Fold 5	Mean	Std Dev
ViT	93.9	98.6	98.8	96.3	98.0	96.8	2.2
ResNet-50	82.2	88.5	89.1	90.4	86.3	87.3	3.2
Vgg-16^15^	81.0	85.0	86.0	84.5	83.0	83.9	1.7
Fundus-DeepNet^8^	85.0	90.0	91.0	89.5	88.0	88.7	2.4

### Patient level classification performance on SIC and EPC-SIC dataset

Our patient-level evaluation metrics extended beyond accuracy to include precision, recall, F1-score, and the area under the receiver operating characteristic curve (AUROC), providing a holistic assessment of model performance. These metrics were carefully selected to provide a multifaceted assessment of the model’s performance, capturing its ability to accurately classify serous cavity effusion cases while minimizing false positives and false negatives, which is crucial for potential clinical applications. As delineated in the patient-level analysis of Table 2, the vision transformer (ViT) model demonstrated superior performance, eclipsing the ResNet-50 model by nearly 9% in accuracy with a remarkable 98.1% attainment. For context, the Vgg-16 and Fundus-DeepNet models posted accuracies of 83.9% and 86.0%, respectively. Notably, all models achieved a recall of 100%, indicating the successful identification of all positive cases within the SIC cohorts. The precision of the ViT model, at 96.8%, was particularly commendable, signaling a substantially reduced rate of false positives relative to the ResNet-50, Vgg-16, and Fundus-DeepNet models. Moreover, the ViT model’s F1-score-a harmonized metric of precision and recall-was outstanding, and its AUROC score reached the pinnacle of 1.00, signifying exceptional discriminative capacity for distinguishing between positive and negative cases at any classification threshold. These findings underscore the ViT model’s robustness and its superior diagnostic reliability over traditional convolutional neural networks (CNNs) for the pivotal task of SCE classification. This is further corroborated by the model’s perfect AUROC scores of 1.00 on the internal dataset and 0.98 on the external EPC-SIC cohort, as depicted in Fig. 1b,c, underscoring its resilience and generalizability. Such exemplary AUROC values at the patient-level suggest the model’s potential to markedly diminish both false negatives and false positives, which is paramount in clinical settings to avoid missed malignancies and to minimize unwarranted patient anxiety and unnecessary procedures. The capacity of the ViT model to precisely pre-screen cytology slides could thus significantly streamline the diagnostic process for pathologists.

The generalizability of the ViT model was rigorously validated on an external cohort (EPC-SIC), where it sustained high accuracy and surpassed the ResNet-50 model by approximately 3%. While the VGG-16 and Fundus-DeepNet models performed admirably on the external cohort, they trailed the ViT model by margins of 8.1% and 6.4%, respectively. The ViT model’s unwavering high recall rate accentuates its robust generalization capability for SCE screening.

Additionally, the models’ performance on diverse SCE types, as outlined in Table 2, showcased the ViT’s consistent dominance with minimal variance in accuracy. In stark contrast, the ResNet-50, Vgg-16, and Fundus-DeepNet models exhibited notable fluctuations in performance. These patterns affirm the robustness of the transformer-based ViT model in adapting to a variety of effusion presentations, and they suggest that the ViT model is a more dependable option for clinical applications where precision and the ability to generalize are of the essence.

**Table 2:** Patient level classification performance (%) of ViT and CNN models on SIC and EPC-SIC dataset.

Model	Type	SIC				EPC-SIC			
		Acc	P	Re	F1	Acc	P	Re	F1
ViT	Pleural	98.0	96.5	100.0	98.2	95.7	93.3	100.0	96.6
	Ascites	98.1	97.1	100.0	98.5	100.0	100.0	100.0	100.0
	Pericardial	100.0	100.0	100.0	100.0	–	–	–	–
	Total	98.1	96.8	100.0	98.4	97.6	96.2	100.0	98.0
ResNet-50	Pleural	88.1	82.1	100.0	90.2	92.9	89.4	100.0	94.4
	Ascites	94.4	91.7	100.0	95.7	96.5	94.3	100.0	97.1
	Pericardial	83.3	80.0	100.0	88.9	–	–	–	–
	Total	89.4	84.4	100.0	91.5	94.5	91.5	100.0	95.5
Vgg-16^15^	Pleural	82.0	76.0	100.0	86.3	88.0	84.2	100.0	91.3
	Ascites	87.5	84.2	100.0	91.3	91.0	88.9	100.0	94.2
	Pericardial	78.0	74.0	100.0	85.0	–	–	–	–
	Total	83.9	78.3	100.0	87.9	89.5	86.3	100.0	92.8
Fundus-DeepNet^8^	Pleural	85.0	79.0	100.0	88.2	90.0	86.7	100.0	93.0
	Ascites	90.0	87.0	100.0	93.2	93.0	90.7	100.0	95.2
	Pericardial	80.0	76.5	100.0	86.7	–	–	–	–
	Total	86.0	80.9	100.0	89.7	91.7	88.4	100.0	94.0

**Figure 2:**
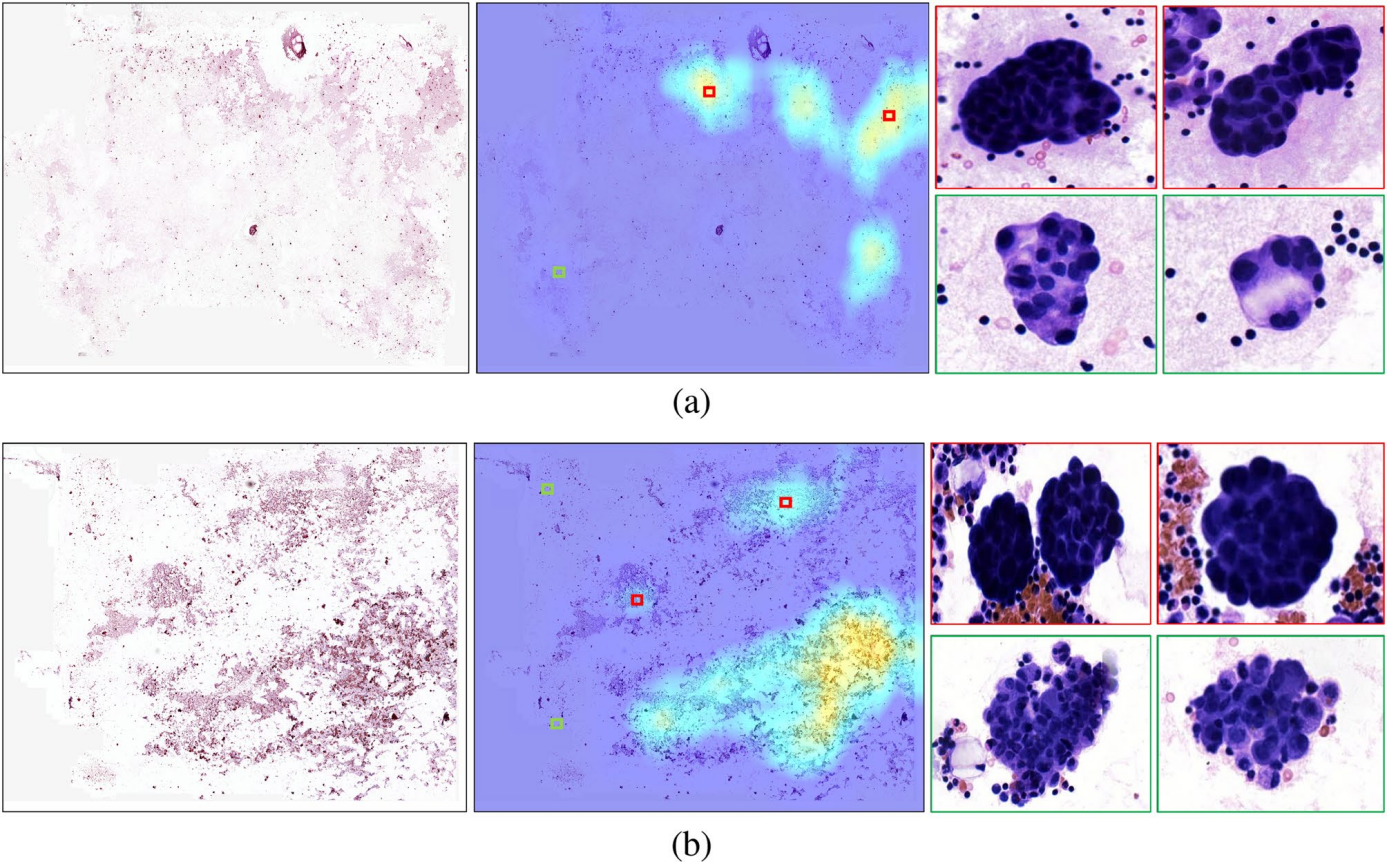
The detection results of two smear cases, denoted as (**a**) and (**b**). For both cases, the original image, heat map, and representative regions are displayed from left to right. The heat maps highlight higher-risk regions for the presence of malignant cells. (**a**) A case with sparsely distributed detection targets, where the highlighted regions were confirmed as positive cell clusters (as indicated by the red boxes), the cell clusters outside the highlighted regions were identified by cytologists as proliferative mesothelial cell clusters (as shown by the green boxes). (**b**) A case with more concentrated detection targets, and we selected highlighted regions outside of the concentrated region to confirm the presence of positive cell clusters (as shown by the red boxes). However, we also observed a large number of clustered cells outside of the highlighted region, which was confirmed by cytologists to be proliferative mesothelial cells (as shown by the green boxes).

### Visualization of smear cases examination results

The examination results of serous cavity effusion smears are visualized using heat maps. These heat maps employ a sliding window technique to scan the image, providing a detailed visual representation of the areas identified by the model. When a particular image block is detected as positive by the model, it is highlighted with a deeper color intensity, indicating a higher likelihood of malignancy. As depicted in Fig. 2, provide a clear visual representation of the model’s diagnostic accuracy in identifying malignant cells within serous cavity effusion smears. In the case illustrated in Fig. 2a, the model adeptly highlights sparsely distributed malignant cell clusters, as indicated by the red boxes. These clusters are accurately differentiated from the benign proliferative mesothelial cells, marked by green boxes, showcasing the model’s ability to discern subtle differences in cell morphology.

In a more challenging scenario shown in Fig. 2b, where malignant cells are densely clustered, the model maintains its precision. It successfully identifies and emphasizes the malignant regions (red boxes), while accurately excluding the nearby proliferative mesothelial cells (green boxes) from the high-risk areas. This level of detail in the heat maps demonstrates the model’s nuanced understanding of the cytological features associated with malignancy, reinforcing its value in assisting pathologists with accurate and reliable diagnoses.

## Discussion

Effusions are a common type of non-gynecological sample collected in clinical practice for both therapeutic and diagnostic purposes^24^. Cytologists are tasked with screening the effusion fluid for malignant cells. Unfortunately, the detailed screening of cytology is time-consuming and labor-intensive. Missed diagnoses can lead to treatment delays and even medical disputes. To address this issue, it is worth exploring new auxiliary diagnostic measures, including the application of artificial intelligence technology, to reduce the workload of doctors and improve diagnostic efficiency.

Recent studies have shown that deep learning models can improve diagnostic accuracy in body fluid cytology detection, particularly for pleural or ascites effusions^15,25^. However, the applicability of these models across different sites is unclear. Moreover, the performance of deep learning in multi-serous cavity effusions has not been explored. We chose to include multi-serous cavity effusions in our study due to several factors. First, these types of effusions present common challenges for cytologists, particularly for junior cytologists, such as the interference of proliferative mesothelial cells on diagnosis^26^. Second, there are significant histological and morphological differences in malignant lesions from different serous cavity effusions^17^. Therefore, it is necessary to train a more universal model that can accurately screen for malignant cells in multiple serous effusions.

This study proposed and assessed a deep-learning-based classification framework for multiple serous cavity effusion screening on two independent patient cohorts. We compared transformer-based (ViT) and CNN-based (ResNet-50) architectures as the classification models of the proposed framework for the patch-level prediction, followed by an aggregation strategy to generate the patient-level predictions. The proposed classification framework with the ViT model achieved superior performance on patch-level and patient-level evaluation of the SIC dataset, with AUROC scores of 0.99 and 1.00, respectively, indicating a high degree of accuracy in identifying malignant effusions. Moreover, it maintained a relatively high performance on the external patient cohort (EPC-SIC), with an AUROC score of 0.98. Especially, the patient-level recall values of the proposed classification framework on both SIC and EPC-SIC are both 100%, suggesting its potential for use in clinical settings.

Traditional body fluid cytology smears often have a large area, with sparse or unevenly distributed detection targets. This presents a challenge for cytologists, who must scan the entire slide without missing any fields. Transformer-based models have been shown to capture global features more efficiently^22^, making them more practical for analyzing large-scale images with sparse targets.

The case study of two smear samples is shown in Fig. 2. The classification heat maps can highlight positive regions, making it easier for cytologists to focus on sparsely distributed positive cells. Additionally, overlapping or excessively deep staining of cells in some cytology smears can obscure cell structures, making it challenging for even experienced cytologists to make accurate judgments based solely on visual cues. In contrast, the transformer-based model has the advantage of automatically capturing more fine-grained features in the smear images^22^. Consequently, the proposed framework has the ability to distinguish between negative and positive cell clusters.

**Figure 3:**
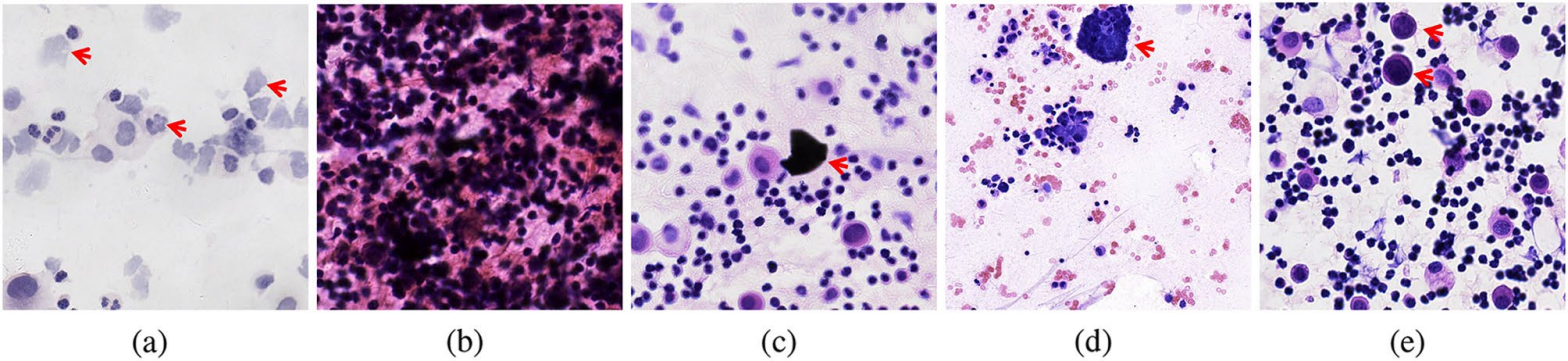
These images show examples of predicted positive patches detected in false-positive cases. Cases that are difficult to differentiate (**a**) and Cases with artifacts (**b**), (**c**) can cause false-positive predictions. Two cases with atypical cells arranged in clusters, or single cells as shown in (**d**) and (**e**) were reinterpreted and confirmed as positive cells by senior cytologists, suggesting that the cases were missed at the initial diagnosis.

Our framework successfully identified all positive cases on both SIC and EPC-SIC datasets. However, there were still a small number of false-positive cases (10 in total). Our error analysis revealed that these cases can be categorized into two types: cases with artifacts and cases that are difficult to differentiate. Figure 3 shows a selection of representative cases, including two cases that exhibit both artifacts and difficult-to-differentiate regions.

Seven of the false-positive cases were attributed to various types of artifacts, including contamination caused by improper processing and very thick smears, as illustrated in Fig. 3b,c. Therefore, it is crucial to ensure proper specimen processing and appropriate quality control^27^. Similar to automated quality assessments on digital histopathology slides^28^, additional quality control measures are necessary for digital smear pictures. The remaining five false-positive cases showed degenerative mesothelial cells, which can lead to false positive results due to degenerative changes such as nuclear hyperchromasia^5^. This is also a pitfall for cytologists, as shown in Fig. 3a.

Interestingly, two senior cytologists confirmed two positive cases from our detected results, as shown in Fig. 3d,e. This indicates that our proposed framework has great potential to assist cytologists in identifying positive cells, which is particularly valuable for medical units with a shortage of experienced cytologists.

Briefly, our study stands out for several reasons: firstly, we evaluated the superior performance of transformer-based models with high performance with an AUROC of 0.99, outperforming traditional CNN-based models by a large margin. Secondly, we tested the proposed classification framework on an external patient cohort, demonstrating its generalization ability and establishing a powerful baseline for future research. Thirdly, our study included three major types of effusions: pleural, ascites, and pericardial, which could increase the clinical application scenarios of the model. Lastly, we demonstrated the clinical-grade classification performance of the proposed framework with a recall of 100%. The right use of heat maps to highlight suspicious positive areas helps cytologists quickly focus on suspicious positive targets. Our model has the potential to screen the accumulation of fluid in the body cavity, similar to how cervical cytology TCT samples are screened^10–12^. This would help cytologists save time by reducing the need for manual review and screening of images.

The theoretical implications of our research are twofold. First, we have demonstrated that transformer-based models, specifically ViT, can effectively handle the complexity of cytology images, which are characterized by large areas and sparse distribution of relevant features. This finding expands the understanding of how self-attention mechanisms can be harnessed in medical image analysis. Second, our work highlights the importance of developing universal models capable of generalizing across various types of serous effusions, which is a step forward in the field of computational pathology.

From a practical standpoint, our study offers a significant contribution to the field of cytology by providing a robust and accurate tool for the screening of serous cavity effusions. The high recall rates achieved by our model ensure that all positive cases are identified, which is critical for patient care. Furthermore, the use of classification heat maps as an assistive tool for cytologists can potentially reduce the workload and improve the diagnostic workflow.

One limitation of our study is the relatively small patient cohort. Future work should include expanding the sample size to further validate our findings. Another limitation is the low amount of pericardial cavity effusions in our data sets. Pericardial effusion accounts for only 11% of all body cavity effusions in the literature. To solve this problem, it is necessary to collect data from more institutions. Additionally, more detailed studies of pleural and ascitic fluid may be conducted to help pathologists determine the type of malignant cells and the histological source of metastatic malignant tumors. This will enable the algorithm to provide a more effective auxiliary tool for cytological detection and diagnosis of body cavity effusion.

Our future research work will focus on the following aspects. First, we will expand our research on cross-modal data. In addition to conventional smears, we will further incorporate liquid-based cytology and cell blocks data. Specifically, we will focus on immunohistochemical staining and combined analysis to enhance the ability of hematoxylin and eosin (H &E)-stained slides in identifying malignant cell origins. Secondly, we will conduct research at the cellular level to predict genetic alterations or targeted therapies for malignant tumor cells. This research will be particularly valuable for selecting treatment plans for late-stage cancer patients. It will provide more cost-effective diagnostic methods and guidance for clinical medication^17,26,29^.

In conclusion, our study underscores the potential of transformer-based models to enhance diagnostic accuracy in serous cavity effusion screening and offers a promising direction for future research in the field.

## Methods

### Data collection

We collected a total of 161 cases from patients who underwent drainage of the serous cavity between 2021 and 2022 at the First Affiliated Hospital of Xi’an Jiaotong University. In our study, we conducted a search in the pathology department’s information system, retrieving cases based on both anatomical location and diagnostic keywords. This dataset includes multiple types of serous cavity effusions, such as pleural fluid, ascites, and pericardial fluid. The detailed data statistics are shown in Table 3. In accordance with the defined criteria^30^, we classified malignant cells and atypical cells as positive samples. Cases with no detection of malignant cells were considered negative. Among the collected cases, 69 were negative and diagnosed with benign serositis. Smears were stained with H &E and digitized using IBL500 scanners (LBP Medicine Science & Technology Co., Ltd., Guangzhou, China) at 40$\times$ magnification (0.345 $\upmu$m/pixel). To confirm the final pathological diagnosis of each case, all positive cases in this study cohort were reviewed by two senior cytologists. Additionally, we collected an external patient cohort of 127 cases from Shaanxi Provincial Cancer Hospital. Our study was conducted in accordance with the ethical principles outlined in the Helsinki Declaration for medical research^31^ and received approval from the Ethics Committee of the First Affiliated Hospital of Xi’an Jiaotong University (XJTU1AF2022LSK-308). Informed consent was waived after approval from the Ethics Committee of the First Affiliated Hospital of Xi’an Jiaotong University.The data collected from patients were de-identified to ensure the protection of their privacy and do not contain any personal health information or identifiable labels.

**Table 3:** The detailed statistics of SIC and EPC-SIC datasets.

Type	SIC				EPC-SIC		
	Pleural	Ascites	Pericardial	Total	Pleural	Ascites	Total
Patient							
Positive	55	33	4	92	42	33	75
Negative	46	21	2	69	28	24	52
Total	101	54	6	161	70	57	127
Patch							
Positive	1281	354	171	1806	–	–	–
Negative	2243	540	247	3030	–	–	–
Total	3625	793	418	4836	–	–	–

### Data annotation

The smear slides of the SIC dataset were annotated by two pathologists from the Department of Pathology at Xi’an Jiaotong University and Shaanxi Provincial Cancer Hospital. Both pathologists had over 15 years of experience in surgical pathological diagnosis. To annotate the slides, we used bounding boxes to outline regions of interest (ROIs) for both positive and negative targets. The OpenHi Digital Pathological Annotation Platform^32^ was used for this purpose. Any uncertain or controversial positive cells were reviewed by a senior cytologist to ensure accurate annotation. In order to improve the accuracy of the annotations, we have established the following principles for annotating positive and negative cases: (1) positive annotation: positive annotations should include malignant cells, which are indicative of cancerous growth. Positive regions may also contain negative cells, such as mesothelial cells and inflammatory cells. These cells should be included within the positive annotation as they can coexist with malignant cells. (2) Negative annotation: negative annotations should strictly exclude any presence of positive cells or components indicating malignancy. The negative regions should only contain normal, non-malignant cells, and any potential benign abnormalities if present.

**Figure 4:**
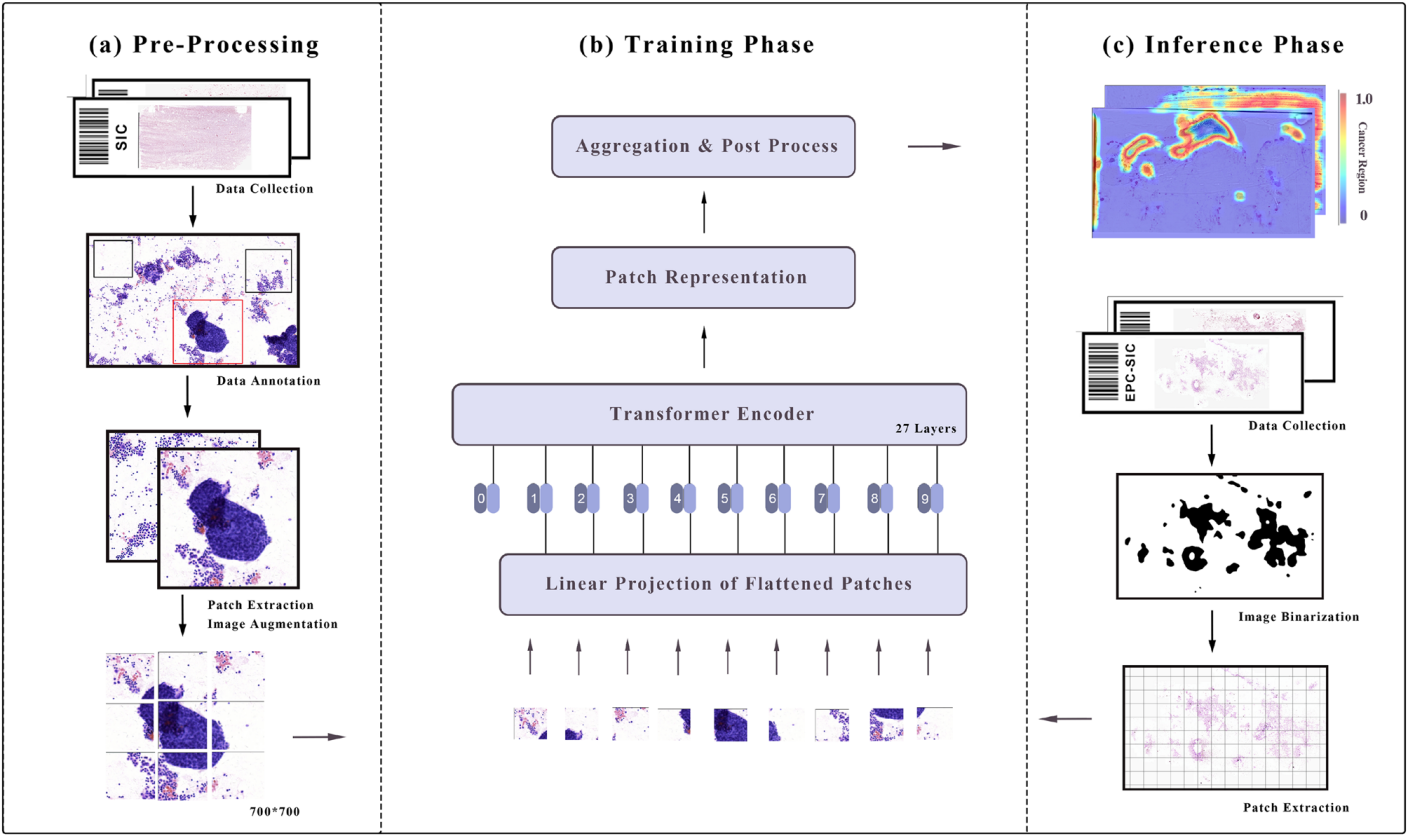
Overview of the proposed transformer-based classification framework.

### Transformer-based classification framework

Transformers have emerged as a powerful tool for image analysis, leveraging self-attention mechanisms to focus on salient features and learn their contextual significance within an image. This is particularly advantageous in medical imaging, where subtle features are critical for accurate diagnosis. The vision transformer (ViT-B/16) architecture treats an image as a sequence of patches, each of which is fed into a transformer network to extract features. The final output of the network is a classification probability distribution over a set of predefined classes. Our implementation of the ViT model has demonstrated exceptional performance on fine-grained image classification^33^. This is due, in part, to the transformers’ ability to model long-range dependencies in the data^34^, which is critical for capturing subtle features. By leveraging the ViT model’s ability to extract features from patches and model long-range dependencies, we can effectively analyze high-resolution pathological images and accurately classify them based on predefined classes.

The overview of the proposed framework is shown in Fig. 4. It consists of three parts, which are (a) the pre-processing, (b) the training phase, and (c) the inference phase.

**Table 4:** The patch partitioning of five-fold cross-validation in the SIC dataset.

Fold	Negative	Positive	Total
1	449	519	968
2	751	216	967
3	584	383	967
4	627	340	967
5	619	348	967
Total	3030	1806	4836

First of all, due to processing high-resolution pathological images, i.e., Whole Slide Images (WSIs), in their entirety is infeasible due to their substantial size and the limited memory capacity of contemporary computer hardware. We employ a sliding window strategy^35^ to process WSIs at the highest level, producing patch-level images of fixed size. The WSI was pre-processed by extracting patches of size $m\times n$ pixels from the regions of interest (ROIs) identified by pathologists. A total of 4836 patches (Table 4) were generated for training. These patches were then resized to a fixed size of $700\times 700$ pixels, with reflective padding used when necessary to conform to the required input size of the ViT-B/16 model, which has approximately 86 million parameters. In comparison, the ResNet-50 model, which we also evaluated, consists of approximately 25 million parameters. Moreover, to ensure uniformity across the dataset, each patch was normalized to have a zero mean and unit variance. To increase the diversity of the training data, we randomly applied two data augmentation techniques from a list that included random rotation, horizontal or vertical flip, contrast adjustment, color intensity alteration, and horizontal shear. This augmentation process allowed us to introduce variability in the dataset, enabling the model to learn more robust features that could be better generalized to unseen data.

Secondly, to achieve patch-level binary classification, we leverage a pre-trained model on the ImageNet dataset and fine-tune it on the SIC dataset. Each patch is classified using the ViT model, and the final result is obtained through aggregation. The architecture of the ViT model is depicted in Fig. 4b, which consists of patch embedding, transformer encoder layers, and a classification head. The patch embedding layer splits the input image into a grid of patches, which are then flattened and projected into a lower-dimensional embedding space. These embeddings are then fed into a stack of transformer encoder layers, each of which consists of multi-head self-attention and feedforward neural network layers. The self-attention mechanism allows the model to attend to different parts of the input sequence, while the feedforward layers introduce nonlinearity and enable the model to learn complex relationships between the patches^33^. The final output of the transformer encoder layers is a sequence of feature vectors representing the input image, which are then passed through a classification head to obtain a probability distribution over the predefined classes. The classification head consists of a linear layer followed by a softmax activation function.

Then, we employed a five-fold cross-validation strategy on the extracted patches. The distribution of the patch-level SIC dataset is shown in Table 4. The model was trained by the cross-entropy loss function and Adam optimizer. We trained the model for 50 epochs with cosine annealing learning rate^36^. The validation set was utilized to assess the model performance.

Lastly, for the slide-level prediction, we employed binarization and morphological operations to separate the foreground and background of the image, enabling the exclusion of regions without cells during processing while disregarding the white background. For whole slide images, we employed a sliding window approach to extract patches from each WSI, with a step size of 700 pixels. The selected patches are then fed back into the ViT model, which generates a final score for each patch. A threshold is then applied to these scores, and patches with scores above this threshold are classified as negative, while patches with scores below the threshold are classified as positive. To aggregate prediction results across all patch levels, we calculated the number of positive and negative regions in the whole slide image to derive the percentage of each category. If all the patches in a WSI are classified as negative, the WSI is considered negative, while if any patch is classified as positive, the WSI is considered positive. Notably, to enhance the method’s stability, we employed a grid search method to identify the optimal threshold for distinguishing positive regions from negative regions in the validation set of each fold.

## Data Availability

All the datasets used in this work are publicly available, whereas datasets that are generated or analysed during labeling, detection and classification are available from the corresponding author on reasonable request.
